# Application of L-Aspartic Acid-Capped ZnS:Mn Colloidal Nanocrystals as a Photosensor for the Detection of Copper (II) Ions in Aqueous Solution

**DOI:** 10.3390/nano6050082

**Published:** 2016-04-27

**Authors:** Jungho Heo, Cheong-Soo Hwang

**Affiliations:** Department of Chemistry, Dankook University, 152 Yongin-si, Suji-ku, Jukjeon-ro, Gyunggi-do 448-701, Korea; cshwang86@hotmail.com

**Keywords:** ZnS:Mn nanocrystals, water-dispersible, aspartic acid capping, Cu^2+^ ion sensor

## Abstract

Water-dispersible ZnS:Mn nanocrystals (NCs) were synthesized by capping the surface with polar L-aspartic acid (Asp) molecules. The obtained ZnS:Mn-Asp NC product was optically and physically characterized using the corresponding spectroscopic methods. The ultra violet-visible (UV-VIS) absorption spectrum and photoluminescence (PL) emission spectrum of the NCs showed broad peaks at 320 and 590 nm, respectively. The average particle size measured from the obtained high resolution-transmission electron microscopy (HR-TEM) image was 5.25 nm, which was also in accordance with the Debye-Scherrer calculations using the X-ray diffraction (XRD) data. Moreover, the surface charge and degree of aggregation of the ZnS:Mn-Asp NCs were determined by electrophoretic and hydrodynamic light scattering methods, respectively. These results indicated the formation of agglomerates in water with an average size of 19.8 nm, and a negative surface charge (−4.58 mV) in water at ambient temperature. The negatively-charged NCs were applied as a photosensor for the detection of specific cations in aqueous solution. Accordingly, the ZnS:Mn-Asp NCs showed an exclusive luminescence quenching upon addition of copper (II) cations. The kinetic mechanism study on the luminescence quenching of the NCs by the addition of the Cu^2+^ ions proposed an energy transfer through the ionic binding between the two oppositely-charged ZnS:Mn-Asp NCs and Cu^2+^ ions.

## 1. Introduction

Transition metal ion-doped semiconductor nanocrystals have been one of the most attractive research subjects for the last few decades [1,2]. Since they display unique physical, chemical, and optical properties, these materials have often been used in various advanced devices, such as non-linear optics [3] and photovoltaic devices [4] and, more recently, in some advanced biomedical areas for diagnostic or therapeutic studies [5,6]. Among the most well-known examples, first-row divalent transition metal ion-doped ZnS nanocrystallites, such as ZnS:Mn [7] and ZnS:Cu [8], have gained special interest due to their high luminescent efficiency and thermal stability, which are essential properties required for commercial electro-luminescence devices [9]. There have been many published articles regarding preparation methods of such materials. However, these methods often require very high temperatures and pressures, and even the use of biologically toxic chemicals [10,11,12].

Certain semiconductor nanocrystals such as CdSe/ZnS [13] and InP/ZnS [14] core-shell quantum dots have been developed as fluorescent labeling agents in biological systems. However, critical problems such as high toxicity and low solubility in water when they were directly used *in vivo* have emerged. In this regard, there have been several papers published regarding the attempt to synthesize water-soluble semiconductor nanocrystals by the modification of their surfaces with polar organic molecules [15]. Among these, thioglycolic acid [16,17] and conventional amino acids [18,19] were found to be effective capping agents in the synthesis of water-soluble and non-toxic semiconductor nanocrystals. However, the ligand-dependent surface properties of water-dispersible nanocrystals are not yet fully understood.

More recently, a novel application of water-soluble semiconductor nanocrystals as selective and sensitive photo-sensors for the detection of certain metal ions in aqueous solution has gained great interest due to their significant effects on human health [20,21]. However, the most commonly used luminescent quantum dots, such as CdS and CdSe, contain environmentally hazardous components. Therefore, the development of a novel type of semiconductor nanocrystal having no biological toxicity and high colloidal stability in water is needed for such applications. Previously in our lab, the synthesis and surface characterization of water-dispersible ZnS:Mn nanocrystals by capping with mercaptoacetic acid (MAA), and their application as a photosensor for the detection of Zn^2+^ and Cd^2+^ ions in aqueous solution, have been reported [22].

In the present study, water-dispersible ZnS:Mn NCs were prepared by capping the surface with conventional L-aspartic acid molecules, which was selected as a capping agent due to its structural advantage for the study aim. An aspartic acid possesses two carboxylic acid moieties that can bind to both the surface of the ZnS:Mn NCs and the target metal ion at the same time. The surface charge of the ZnS:Mn-Asp NCs was determined by an electrophoretic light scattering method. For the surface characterization of the ZnS:Mn-Asp NC product, the degree of aggregation in water was measured using a hydrodynamic light scattering method. Finally, the ZnS:Mn-Asp NCs with a negative surface charge was presumed to be suitable for further coordination to transition metal ions in aqueous solution. This was intended to show the potential of the ZnS:Mn-Asp NCs as a specific transition metal ion photosensor.

## 2. Results and Discussion

### 2.1. Characterizations of the ZnS:Mn-Asp NCs

**Figure 1:**
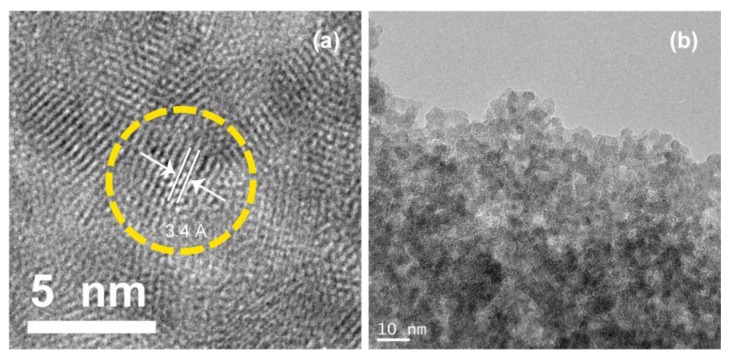
High resolution-transmission electron microscopy (HR-TEM) images of the ZnS:Mn-Asp nanocrystals (NCs). Asp: L-aspartic acid.

The average particle size of the ZnS:Mn-Asp NCs was measured from the HR-TEM images provided in Figure 1. Even though it was difficult to find a clear image of the discrete individual particles, we enlarged the picture as much as possible and measured diameters of 30 identifiable spherical shaped particles, along the fringe images, to calculate the average particle size for the ZnS:Mn-Asp NCs. The shape of most particles was fairly close to spherical, and the average particle size was 5.25 nm. Little agglomeration between the particles was observed due to evaporation of the water/alcohol solvent during the sample preparation procedures. However, the appeared distinct lattice planes in the fringe images, which are spacing to each other about 3 Å distance, indicates that the ZnS:Mn-Asp NCs were consisted of single crystals rather than adducts of poly-crystalline mixtures. Precise measurements of the elemental composition of the prepared ZnS:Mn-Asp NCs and the doping concentration of the manganese (II) ions (Mn^2+^) were performed using inductively-coupled plasma-atomic emission spectrometry (ICP-AES). Three trials of sample measurements revealed that the average elemental proportion of Mn^2+^ ions relative to the ZnS parent crystals was 1.7%. The doping concentration of the Mn^2+^ ions in the ZnS parent crystals was originally intended to be 1%–2%, which has been reported to be the optimum for PL efficiency in previously-known ZnS:Mn NCs [23,24].

**Figure 2:**
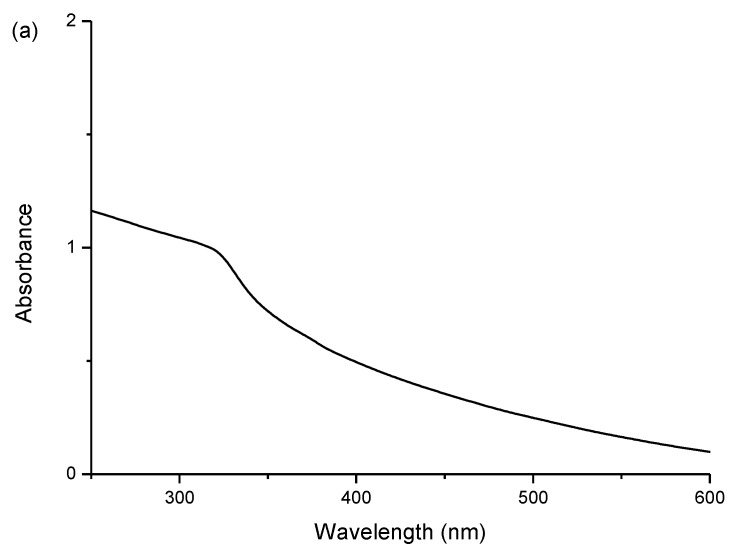

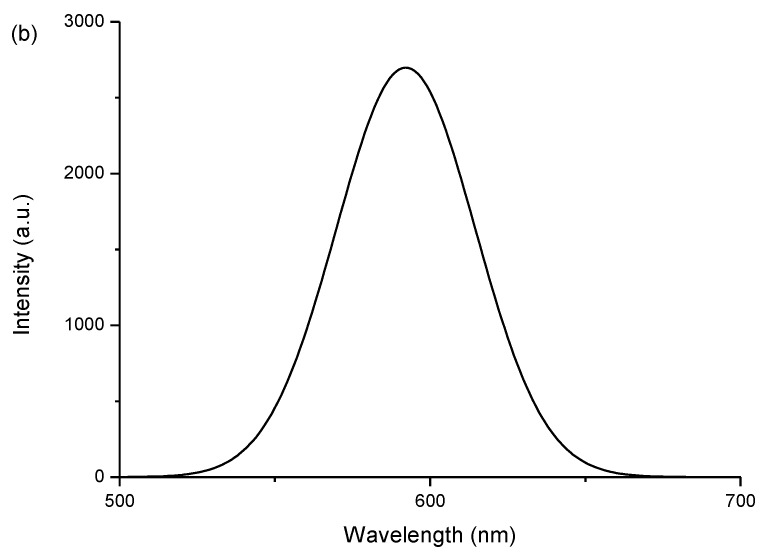
(**a**) Ultraviolet-visible (UV-VIS) absorption spectrum; and (**b**) room temperature solution photoluminescence (PL) emission spectrum of the ZnS:Mn-Asp NCs. (λ_max_ of the fluorescence peak was 590 nm).

The optical properties of the as prepared ZnS:Mn-Asp NCs were investigated using UV-visible (UV-VIS) and photoluminescence (PL) spectroscopy. Figure 2a,b show the obtained UV-VIS absorption and PL emission spectra of the ZnS:Mn-Asp NCs, respectively. The PL spectrum showed a broad emission peak appeared at 590 nm, which was obtained by adjusting the excitation wavelengths of the light source at the same as UV-VIS absorption peak of the ZnS:Mn-Asp NCs (320 nm). The major absorption peak shown in the spectrum probably resulted from the well-known band-to-band absorption in the ZnS host lattice [25], and the increased band gap of the ZnS:Mn NCs (3.87 eV) compared with that of the bulk ZnS:Mn solid (3.54 eV) was likely due to the well-known quantum confinement effect [26]. The yellow-orange light emission at 590 nm was attributed to the ^4^T_1_–^6^A_1_ transition of the dopant (Mn^2+^) ions [27]. The energy transfer between the ZnS host and the Mn^2+^ activator can be significantly interrupted when the states of the surface defects are located close to the conduction band of the ZnS, which can reduce the intensities of the emissioin and enlarge the Stoke shifts. The PL efficiency of the ZnS:Mn-Asp NCs was measured and calculated following the method reported by Williams *et al*. [28]. This method includes the calculation of a relative quantum yield by comparison with that of a commercial organic dye standard, a 0.1 M solution of quinine sulfate dissolved in sulfuric acid (purchased from Fluka) in this experiment [29], of which the reported emission wavelength and absolute quantum yield are 550 nm and 0.546, respectively (22 °C). The excitation wavelength for the maesurment of the comparing dye standard was adjusted at the same value as the ZnS:Mn-Asp NCs, which was obtained from the UV-VIS absorption spectrum of the NCs. As a result, the calculated relative PL efficiency was 9.81%, which is slightly higher than that of other amino acid-capped ZnS:Mn NCs measured in water [30].

**Figure 3:**
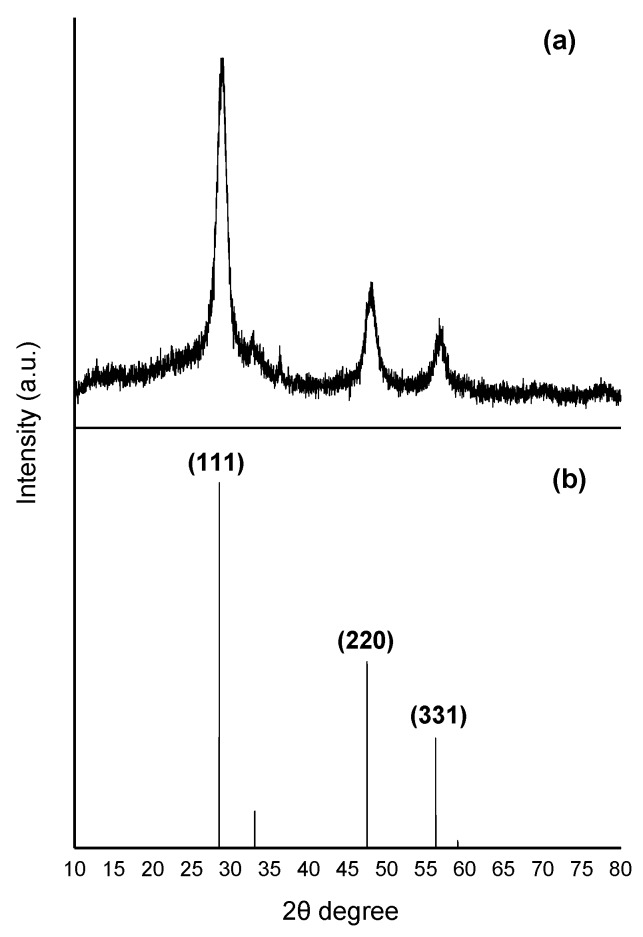
X-ray diffraction (XRD) patterns of: (**a**) ZnS:Mn-Asp NCs; and (**b**) ZnS bulk solid in a cubic zinc-blende phase (JCPDS 05-0566) as an internal reference.

In Figure 3, the wide-angle X-ray diffraction (XRD) pattern obtained from the ZnS:Mn-Asp NCs is presented. Among the most broad peaks in the diagram, three identifiable peaks: (111), (220), and (311) clearly indicated that the ZnS:Mn-Asp NCs are in the cubic zinc-blende phase (JCPDS 05-0566) [31]. In addition, we also compared the particle size of the ZnS:Mn-Asp NCs calculated by applying the Debye-Scherrer equation using the XRD peak data with that directely measured from the HR-TEM image in Figure 1 [32]. Taking the measured values of full width at half maxima (FWHM) of the selected XRD peaks, we calculated an average particle size for the ZnS:Mn-Asp NCs as 5.41 nm, which showed relatively good agreement with that measured from the HR-TEM image.

**Figure 4:**
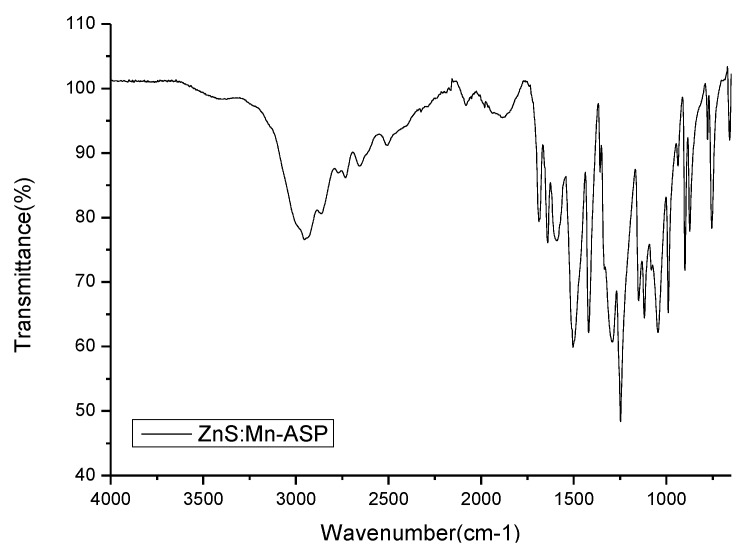
Fourier transform infrared (FT-IR) spectrum of the ZnS:Mn-Asp NCs.

The amino acid (Asp) molecules attached to the surface of the ZnS:Mn NCs were characterized by Fourier transform infrared (FT-IR) spectroscopy. Figure 4 presents the FT-IR spectrum of ZnS:Mn-Asp NCs. Table 1 presnts the obtained peak data and their assignments. These specific vibrational modes were assinged by comparison with previously-reported assignments of the FT-IR spectrum of free L-aspartic acid (Asp) associated with a computational calculation method [33,34]. In the spectrum, the overall peaks obtained from the surface capping ligands (Asp molecules) were slightly red-shifted compared with those of the neat or uncoordinated Asp molecule, since some of the vibrational modes were restricted by attaching to much heavier transition metal ions (Zn^2+^ or Mn^2+^) on the surface of the nanocrystal lattice [35,36]. The most characteristic peaks of the NC-coordinated Asp molecule were the two C=O stretching peaks appearing at 1717 and 1641 cm^−1^. The former carbonyl stretching peak is similar to that for free Asp. The latter carbonyl peak was not found in the free Asp, and the intensity of the peak was much smaller than the former carbonyl stretching peak. The latter peak was assigned as a carbonyl group attached to a metal ion (Zn^2+^ or Mn^2+^) on the surface of the NCs, since the wave number of the peak was very close to the previously-reported Ti^2+^-coordinated Asp molecule [37]. Therefore, according to these assignments, one of the carbonyl groups of the Asp molecule was attached to the nanocrystal surface, while the other carboxyl group and the amine end remained uncoordinated to impart a hydrophilic nature to the NCs. In addition, any uncoordinated or unreacted Asp molecules were removed by centrifuging and washing several times with a cold alcohol/water solution. As a result, the peaks from any free Asp molecules could be removed from the obtained FT-IR spectrum for the ZnS:Mn-Asp NCs. Finally, the majority of peaks appearing in the lower region, from 400 to 1000 cm^−1^, can be assigned as mixed banding modes of C–C, and C–O moieties of the NC-coordinated Asp molecules.

### 2.2. Surface Properties of the ZnS:Mn-Asp NCs

To investigate the surface characteristics of the ZnS:Mn-Asp NCs in water, the surface charge and degree of aggregation were determined by the electrophoretic light scattering method [38] and hydrodynamic light scattering method [39], respectively. As a result, the zeta potential of the NCs at ambient temperature was obtained as −4.58 mV. Considering the acidic nature of aspartic acid, the negative surface charge of the ZnS:Mn-Asp NCs can be explained by the fact that the protons originally belonging to the carboxylic ends (COOH) of the Asp molecules were ionized in the water. Therefore, their conjugate base ion moieties (COO^−^) on the NC surface actually caused the observed negative surface charge. However, it should also be noted that the carboxyl end is not so strongly acidic in water compared with halide acids; thus, not all of the protons in the carboxyl (–COOH) end were ionized in this environment. A very similar phenomenon was observed for the previously-mentioned MAA-capped ZnS:Mn NCs, in which the reported zeta potential of the NCs was −22.38 mV under the preparation conditions of pH 2 [22]. A plausible explanation for obtaining a much smaller negative charge value for the ZnS:Mn-Asp NCs is that a portion of the ionized protons were attracted to the neighboring amine (NH_2_) end of the Asp molecules to form NH_3_^+^ moieties, in order to cancel out the negative charge caused by the (COO^−^) ends. However, the two opposite charges did not perfectly cancel each other out, since the preparation pH conditions were far below from the known isoelectric point of Asp molecules (pI = pH 5.74, CAS 617-45-8).

Secondly, the measured particle size distribution of the ZnS:Mn-Asp NCs in water indicates that the NCs formed various sized aggregates in water. The calculated average size of the agglomerates was 19.8 ± 2.78 nm. These agglomerates likely formed from the intermolecular interaction (mostly hydrogen bonding) between the COO and NH groups in the capping aspartic acid molecules on neighboring ZnS:Mn NCs, since the NCs were originally observed to form 5 nm-sized particles in the solid state according to the previously shown HR-TEM image and the Debye-Scherrer’s XRD calculation. Similar intermolecular attractions between the capping molecules on the NC surface for L-glycine and L-alanine capped ZnS:Mn NCs have been reported [18], in which the intermolecular interaction (also mainly hydrogen bonding) between the amino acid molecules caused the formation of huge agglomerates of the corresponding ZnS:Mn-aminoacid nanocrystals (from 250 nm to a few micrometer in size) in water. However, the degree of aggregation for the ZnS:Mn-Asp NCs was relatively low in water compared to other aminoacids capped ZnS:Mn NCs, which was probably due to the electrostatic repulsion between the negatively-charged NC surfaces. A similar phenomenon has been seen with ZnS:Mn-MAA NCs prepared at pH 7, with the formation of much larger agglomerates in water than at pH 2, in which the repulsion between the NCs was significantly reduced [40].

### 2.3. Photosensor Activity of the ZnS:Mn-Asp NCs

**Figure 5:**
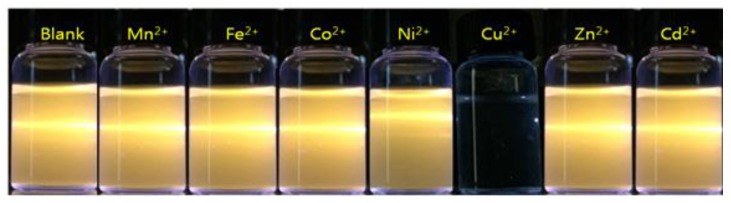
Light scattering image of the ZnS:Mn-Asp NCs upon addition of transition metal ions, taken under irradiation of He-Cd laser (325 nm) light. (‘Blank’ refers to ZnS:Mn-Asp NCs only).

Since the negatively-charged NCs were presumed to be suitable for further coordination by positively charged transition metal ions, the ZnS:Mn-Asp NCs were tested as a cation photosensor for Mn^2+^, Fe^2+^, Co^2+^, Ni^2+^, Cu^2+^, Zn^2+^, and Cd^2+^ ions. As a result, fast and exclusive luminescence quenching was observed upon the addition of an aqueous solution containing Cu^2+^ ions, as shown in a light scattering picture in Figure 5.

**Figure 6:**
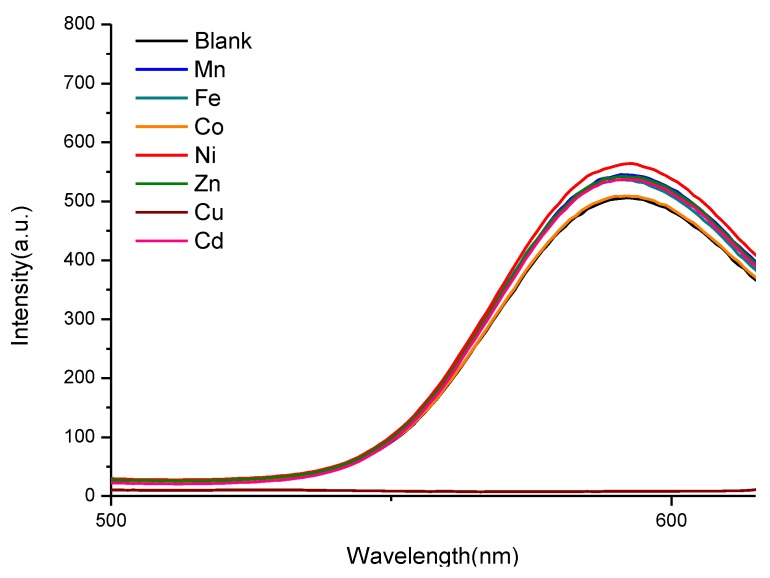
Emission (PL) spectra of the ZnS:Mn-Asp NCs following the addition of divalent transition metal ions. The emission peak of the ZnS:Mn-Asp-Cu (brown) was exclusively diminished. (‘Blank’ refers to no addition of transition metal ions).

Additionally, Figure 6 presents the photoluminescence (PL) emission spectra following the addition of divalent transition metal ions to the ZnS:Mn-Asp NC-containing solution. As can also be seen in these spectra, the strong emission peaks of the ZnS:Mn-Asp NCs remained for most metal ions, while Cu^2+^ ions caused almost complete fluorescence quenching. This unique result led us to preliminarily conclude that the ZnS:Mn-Asp NCs can be used as a selective metal ion photosensor for Cu^2+^ ions in water. The sensing limit of the added molar concentration of metal ions ([M]) was 8.2 μM over 1.0 mg/L ZnS:Mn-Asp NCs. Both Figure 5 and Figure 6 were obtained by adjusting the concentrations of [Cu^2+^] at 15 µM over 1.0 mg/L of the ZnS:Mn-Asp NCs to make sure that the orange-yellow fluorescence light of the NCs was completely disappeared by the addition of the Cu^2+^ ions, while that of other transition metal-NC mixture samples were almost unchanged. At this concentration, the measured [Cu^2+^]/[NCs] ratio was 0.32, according to an additional inductively coupled plasma atomic emission spectroscopy ( ICP-AES) analysis.

**Figure 7:**
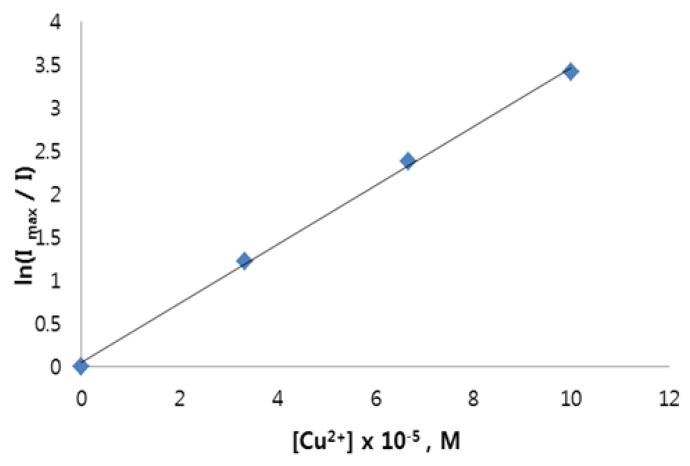
A linear fitting diagram showing the modified (natural log-dependent) Stern-Volmer relationship between the ZnS:Mn-Asp NCs and the added Cu^2+^ ions. (*R*^2^ = 0.9989, and the quenching rate constant k = 3.14 × 10^4^ M^−1^).

Previously, we have reported a similar study of the use of surface-modified ZnS:Mn NCs with a different ligand (mercaptoacetic acid, MAA) for the detection of heavy transition metal ions in aqueous solution [22]. However, their fluorescence quenching effects were totally different from those of the ZnS:Mn-Asp NCs. The addition of transition metal ions to the ZnS:Mn-MAA NCs caused the yellow-orange emission light to remain only when Zn^2+^ and Cd^2+^ ions were added, while other metals caused almost complete fluorescence quenching. The proposed mechanism for these results is that the added metal ions were coordinated by the MAA ligands belonging to neighboring ZnS:Mn NCs to form a metal-bridged nanocrystal composite. Subsequently, additional energy transfer between the nanocrystals and the coordinated metal ions caused quenching of the luminescence from the ZnS:Mn NC parents. Since the ZnS:Mn-Asp NCs showed different metal ion selectivity of luminescence quenching, one can speculate that the quenching mechanism must be different from that of the ZnS:Mn-MAA NCs. In fact, the results seen with the ZnS:Mn-Asp NCs were quite similar to those for the mercaptopropionic acid (MPA)-capped CdTe nanoparticles, which showed an exclusive fluorescence quenching effect for Cu^2+^ ions in aqueous solution [41]. The proposed quenching mechanism was ionic binding of the Cu^2+^ ions with the CdTe-MPA NCs according to modified Stern-Volmer kinetics [42,43]. We also evaluated the degree of decrease in PL intensity as the concentration of Cu^2+^ ions gradually increased. As a result, the obtained data fit well into the modified Stern-Volmer equation: ln(*I*_max_/*I*) = 1 + k[Q], where k is the phosphorescence quenching constant, which is related to the quenching efficiency of the quencher, and [Q] is the concentration of the quencher ([Cu^2+^]). The resulting plot, Figure 7, shows a linear relationship between 0 and 100 µM (*R*^2^ = 0.9989). The obtained quenching rate constant value (k = 3.14 × 10^4^ M^−1^) was calculated from the slope of the line. The plot of ln(*I*_max_/*I*) as a function of [Cu^2+^] indicates that the luminescence quenching of the ZnS:Mn-Asp NCs was probably due to the ionic binding between the ZnS:Mn-Asp NCs and the added Cu^2+^ metal ions. For these experiments, the molar concentration of the ZnS:Mn-Asp NCs was fixed at 200 mg/L, and the addition concentration range of [Cu^2+^] was carefully selected to avoid complete luminescence quenching in order to obtain proper ln(*I*_max_/*I*) ratio values. There are several known fluorescence quenching mechanisms for colloidal semiconductor nanocrystals upon addition of various transition metal ions such as energy transfer, complex formation, and ionic binding quenching processes in the literature [44]. The complex formation and ionic binding quenching processes are often referred to as static and dynamic quenching, respectively. A tremendous amount of kinetic studies regarding those fluorescence quenching mechanisms were carried out, and it was suggested that obtaining a linear relationship between the luminescence intensity changes and the variation of the added metal ion concentration using a Stern-Volmer equation supports the static (complex formation) quenching mechanism, while the latter (ionic binding) shows a linear fitting in a modified (natural log-dependent) Stern-Volmer equation [45,46]. Based on the suggestions by these kinetic studies, we concluded that the quenching mechanism of the ZnS:Mn-Asp NC upon addition of Cu^2+^ ions is more close to the ionic binding quenching process since we obtained the linear fitting plot of ln(*I*_max_/*I*) *vs.* [Cu] by using the modified (natural log-dependent) Stern-Volmer equation.

**Figure 8:**
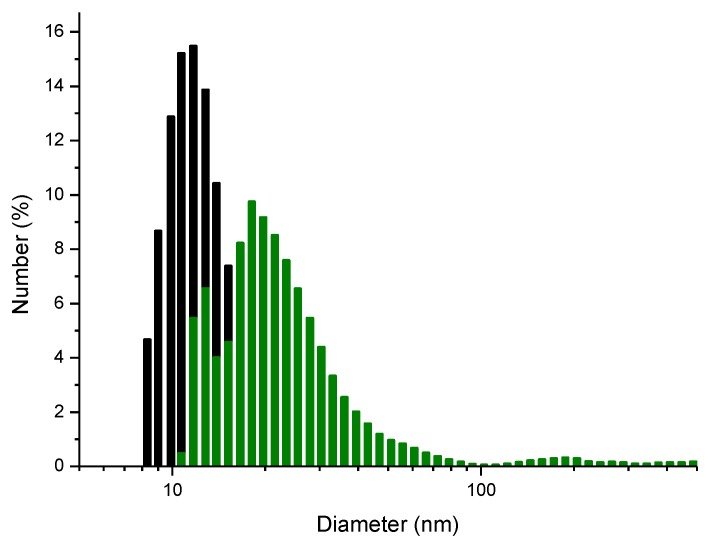
Particle size distribution histograms of the ZnS:Mn-Asp NCs only (black, 19.8 nm in average), and the ZnS:Mn-Asp-Cu adduct (green, 58.4 nm in average) in aqueous solution.

In addition, to support the proposed ionic binding mechanism, particle size distribution diagrams were taken before and after the addition of a small amount of Cu^2+^ ion-containing solution to the ZnS:Mn-Asp NCs. As can be seen in Figure 8, the original ZnS:Mn-Asp NCs formed agglomerates with an average size of 19.8 nm in water, however, the addition of the Cu^2+^ ion-containing solution caused the formation of larger agglomerates with an average size of 58.4 nm. According to ICP-AES elemental analysis of the ZnS:Mn-Asp-Cu solid mixture, the [Cu]/[NC] ratio was 0.03. For this analysis, the ZnS:Mn-Asp-Cu solid mixture was isolated and washed with a cold water/alcohol mixture to remove any uncoordinated free Cu^2+^ ion salt. Therefore, the copper ions detected by the ICP-AES in the solid mixture probably belong to the NC-Cu adducts.

**Figure 9:**
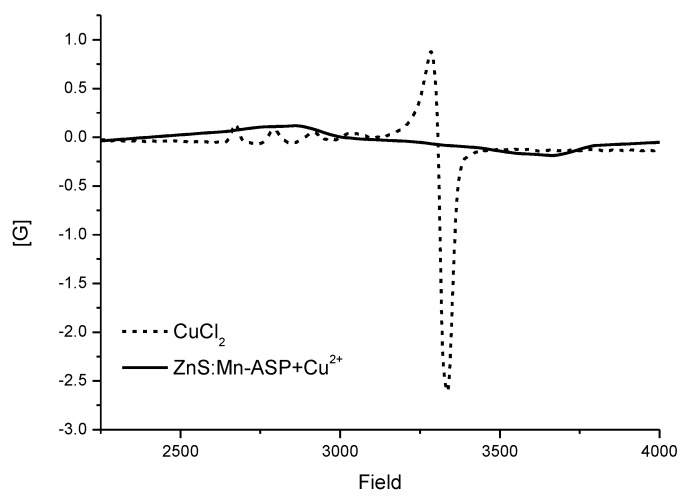
X-band electron paramagnetic resonance (EPR) spectrum of the ZnS:Mn-Asp-Cu adduct (solid) taken at 200 K. The spectrum of a paramagnetic CuCl_2_ (d^9^) solid (dashes) is also presented for comparison.

It has been shown that the NC surface-attached Cu^2+^ ions induced electron transfer from the NCs to cause luminescence quenching by reduction of the copper to the +1 oxidation state [41]. Therefore, we also evaluated the oxidation state of the copper ions after addition to the ZnS:Mn-Asp NCs. As shown in Figure 9, the electron paramagnetic resonance (EPR) spectrum obtained from the ZnS:Mn-Asp-Cu adducts clearly showed a diamagnetic character, indicating that the copper ions were reduced from Cu^2+^ (d^9^) to Cu^1+^ (d^10^). In this experiment, the added amount of the Cu^2+^ ions was carefully controlled to achieve a [Cu]/[Mn dopant] atomic ratio of 250, since the paramagnetic Mn^2+^ ions doped in the ZnS:Mn-Asp NCs and the remaining free (not bound to the NCs) Cu^2+^ ions can also affect the EPR measurement. Thus, paramagnetic peaks for the Mn^2+^ ions became negligible in the EPR spectrum. This result can be considered as direct experimental evidence that the electron transfer process from the NCs to the Cu^2+^ ions causes the luminescence quenching of the NCs. In addition, the exclusive quenching of the ZnS:Mn-Asp NCs by copper ions can be explained by the fact that the reduced Cu^1+^ ions (d^10^ species) can be easily stabilized by relieving the Jahn-Teller distortion, while other metal ions do not have significant energetic motive to induce the electron transfer from the ZnS:Mn-Asp NCs. In addition, the exclusive metal ion (Cu^2+^) quenching selectivity of the ZnS:Mn-Asp NCs can also be explained by applying the Irving and Williams’ calculations of the stability constants of complex formation for various transition metal ions with various poly-dentate ligands [47]. According to the paper, copper (II) ions have an exceptional affinity to the carboxyl ends of the amino acid molecules compared to other divalent transition metal cations. Even though most divalent transition metal ions have some extent of affinities to the carboxylate anion, the aspartic acid molecules attached on the ZnS:Mn-Asp NCs also have a neighboring positive NH_3_^+^ group to strongly repel approaching metal cations. Therefore, only copper (II) ions, which possess the highest affinity to the carboylate anion, can barely bind to induce further electron transfer from the ZnS:Mn-Asp NCs in this condition.

Some other possible quenching mechanisms for the semiconductor nanocrystals upon addition of metal ions have been proposed by several research groups. For instance, Xie *et al.* [48] studied and reported a luminescence quenching mechanism of the bovine serum albumin (BSA) modified CdSe/ZnS quantum dots upon addition of Cu^2+^ ions in aqueous solution. They proposed that copper (II) ions are small enough to penetrate between the bulky BSA capping ligands and they can react with sulfide ions attached on the surface of the CdSe/ZnS quantum dots (QD) to form a CuS crystal on the surface of the QD. Growing of the CuS can decrease the band gap of the QD, which can induce a non-radiative electron-hole recombination process. However, the quenching mechanism of the ZnS:Mn-Asp NCs is obviously different from the CdSe/ZnS QD case because the Cu^2+^ ions bound to the ZnS:Mn-Asp NC surface were reduced to Cu^1+^ ions, according to our EPR measurement, while the CuS crystal should maintain the Cu^2+^ oxidation state to build-up a stable crystal structure on the different crystal surface. Other different luminescence quenching mechanisms have been proposed that the surface-capping ligands can be removed from the NC surface by formation of stable complexes with added metal ions, which also induce huge agglomerations and precipitations of the NCs in water to diminish the fluorescence of the NCs [49]. However, in that case, the original oxidation state of the metal ions should not be changed after complexation since the electron transfer from the NCs to metal ions is impossible in this environment, so this case was also excluded from our explanation for the fluorescence quenching mechanism of the ZnS:Mn-Asp NCs. However, a very similar electron transfer process from NCs to Cu^2+^ metal ions has been reported by Chen *et al.* [50] in the fluorescence quenching study of thioglycerol-capped CdS NCs by Cu^2+^ ions. The authors observed that Cu^2+^ ions attached to the NC surface were completely reduced to Cu^1+^ ions, which facilitated non-radiative recombination of excited electrons in the conduction band and holes in the valence band, in which a kinetic study also showed a similar linear fitting relationship in ln(*I*_max_/*I*) *vs.* [Cu]. Therefore, the most plausible luminescence quenching mechanism for the ZnS:Mn-Asp NCs is that the Cu^2+^ ions were attracted by the negatively-charged ZnS:Mn-Asp NCs, and then bound to the surface of the NCs followed by an electron transition occurred from the NCs to the Cu^2+^ ions to induce the reduction of the Cu^2+^ ions, which directly caused the luminescence quenching by interrupting the electron-hole recombination process at the Mn centers in the ZnS:Mn-Asp NCs at the same time.

Finally, it is notable to mention that the totally different quenching metal selectivity of the ZnS:Mn-Asp NCs compared with the previously reported ZnS:Mn-MAA NCs probably caused by the presence of the neighboring NH_3_^+^ moiety in the aspartic acid molecule. The carbonyl group in the MAA molecule can form a stable complex with an added transition metal cation in water. However, in the aspartic acid molecule, the neighboring NH_3_^+^ group can interrupt to form a stable complex since the protonated amine functional group has the same positive charge with the metal ions in this pH condition. Therefore, the ZnS:Mn-Asp NCs were not able to advance a complexation-electron transfer pathway for the quenching as in the ZnS:Mn-MAA NCs. The important role of the side amine group in aspartic acid, in the formation of a complex with a metal ion, has been shown in several crystal structures containing the [metal-Asp] complexes in the literature, in which the central metal ion was coordinated by the COO and NH ends of the Asp, together, to stabilize the complexes [51,52].

## 3. Experiemntal

### 3.1. Instrumentation

The HR-TEM images shown in the present article were taken by a JEOL JEM 1210 electron microscope (JEOL, Tokyo, Japan). The range of magnification mode and accelerating voltage were 1000 to 800,000 and 40–120 kV respectively. To prepare the samples, ZnS:Mn-Asp NC powder was dried and dispersed in methanol, then it was placed on a carbon-coated Cu grid (300 mesh) followed by drying in a vacuum oven for 20 h. UV-VIS absorption spectra were obtained by using a Perkin Elmer Lamda 25 spectrophotometer (Waltham, MA, USA) which is equipped with a deuterium/tungsten lamp. The solution photoluminescence (PL) spectra were taken by using a Perkin Elmer LS-45 spectrophotometer (Waltham, MA, USA) which is equipped with a 500 W Xenon lamp, a 0.275 m triple grating monochrometer, and a PHV 400 photomultiplier tube. A Rigaku 300 X-ray diffractometer (Rigaku, Tokyo, Japan) equipped with a Cu Kα (1.54 Å) wavelength light source was used to obtain the XRD pattern diagrams. The elemental analyses for the ZnS:Mn-Asp NCs were performed using an Optima-430 (Perkin Elmer, Waltham, MA, USA) ICP-AES spectrometer containing an Echelle optics system and a segmented array charge-coupled device (SCD) detector. The FT-Raman spectra, which were obtained to characterize the surface capping Asp molecules, were recorded using a Bruker FRA106/s spectrophotometer with a resolution of 1 cm^−1^. The reported surface charge and the degree of agglomerations of the ZnS:Mn-Asp NCs in aqueous solution in this article were measured using an ELS-8000 Zeta-PSA spectrometry (Otsuka Electronics, Tokyo, Japan) which is equipped with a 10 mW He/Ne laser light source. Finally, the presented EPR spectrum was obtained using a Bruker X-band CW-EPR (9–9.8 GHz) (ELEXSYS-II E 500, Madison, WI, USA).

### 3.2. Synthesis of the ZnS:Mn-Asp NCs

ZnS:Mn-Asp NCs were synthesized by following a previously reported method of L-valine-capped ZnS:Mn NCs, which involes a formation of [zinc (II) ion-aminoacid] moiety containing reactive intermediate complexes [51]. ZnSO_4_·5H_2_O (1.44 g, 5 mmol) was dissolved in 50 mL water and the mixture was slowly added to a 50 mL aqueous solution containing 10 mmol of L-aspartic acid at 5 °C (an ice-water bath used). The mixture solution was warmed to room temperature standing 1 h of stirring. For nucleation of the seed crystal and doping, MnSO_4_·H_2_O (0.02 g, 0.1 mmol) and Na_2_S (0.40 g, 5 mmol) were dissolved in 20 mL deionized water. The resulting mixture was subsequently added to a flask containing the [Zn-Asp] intermediate complexes under vigorous stirring. The whole mixture solution was refluxed for 10 h, followed by slow cooling to room temperature and the addition of absolute ethanol, which afforded an white-grey precipitate at the bottom of the flask. Finally, the product NC solids were separated by centrifugation and decanting of the water-ethanol supernatant. The solids were transferred and dried for 24 h in a vacuum oven. Detailed experimental data are listed in Table 2.

### 3.3. PL Efficiency Measurements

The PL efficiency of the ZnS:Mn-Asp NCs was measured and calculated by following the same method reported by Williams *et al*. [28]. This method is frequently used to calculate a relative quantum yield by comparison to a standard organic dye [29], a 0.1 M solution of quinine sulfate in H_2_SO_4_ (purchased from Fluka, Seoul, Korea) in this case, of which the fluorescence wavelength and absolute quantum yield are reported to be 550 nm and 0.546, respectively (22 °C). The excitation wavelength used for the commercial organic dye standard was adjusted at the same with the ZnS:Mn-Asp NCs, which was obtained from the UV-VIS absorption spectrum. The emission spectra for both the standard and the ZnS:Mn-Asp NCs were recorded at five different concentrations in aqueous solution. A graph of integrated PL intensity *versus* absorbance for both samples obtained at different concentrations was plotted, and the relative PL efficiency for the ZnS:Mn-Asp NCs was calculated by applying the following equation:

$$\emptyset_{X\;}=\;\emptyset_{\mathrm{ST}}\left(\frac{{\mathrm{Grad}}_{X}}{{\mathrm{Grad}}_{\mathrm{ST}}}\right)\left(\;\frac{\varepsilon_{X}^{2}}{\varepsilon_{\mathrm{ST}}^{2}}\right)$$

In this equation, ∅ represents PL efficiency. The subscript ST and x denote the standard dye and the NCs, respectively. In addition, ‘Grad’ is the gradient from the plot of integrated fluorescence intensity *versus* absorbance, and ‘ε’ indicates the solvent refractive index. In fact, we excluded the solvent factor by using the same solvent (water) for both the standard and the ZnS:Mn-Asp NCs.

## 4. Conclusions

Recently, low-dimensional semiconductor nanocrystals have received attention as a novel type of photosensor material for the detection of certain metal ions in biological systems, as well as in drinking and waste water. Since ZnS:Mn NCs can provide a novel platform to attract transition metal cations to its surface by the proper control of the surface-capping ligands, it can be used for a variety of applications in photo-sensing devices. In the present study, conventional amino acid L-Asp-capped ZnS:Mn NCs were successfully synthesized, and their characteristic surface properties were investigated using spectroscopic methods. In addition, the related physical and optical properties were also investigated. The as-prepared water-dispersible ZnS:Mn-Asp NCs showed remarkable physicochemical properties for further applications in an advanced photosensor device. In particular, in the present study, ZnS:Mn-Asp NCs showed very different photosensor activity compared with other ligand-capped ZnS:Mn NCs, indicating that one can control the selectivity of the ZnS:Mn NCs for certain metal ions simply by changing the surface-capping ligand. This has not been seen in other semiconductor nanocrystals in the literature, making them a unique candidate for various metal ion sensors using a single NC source. In addition, it was also demonstrated in this study that both the degree of aggregation in water and the surface charge of the capping ligand are critical factors to be considered for further application in any commercial device.

## Figures and Tables

**Table 1:** FT-IR data and assignments of ZnS:Mn-Asp NCs. (units in cm^−1^).

ZnS:Mn-Asp	Free L-Asp [33]	Assignments
Not appeared	479	δ(N–H)
Not appeared	525	δ(C–C–O)
658	643	δ(O–C–O)
874	876	ω(C–C)
899	894	ρ(C–C)
1047	1046	ν(C–N)
1152	1153	ρ(N–H)
1248	1257	ν(C–H)
1310	1312	δ(O–H)
1420	1422	ρ(C–H)
1504	1514	ν(OCO) + δ(C–H)
1641	Not appeared	ν(C=O–M)
1717	1736	ν(C=O)
2950	3013	ν(N–H)
3350	3434	ν(O–H, H_2_O)

**(**ν—stretching; ω—wagging; δ—in-plane deformation; ρ—rocking).

**Table 2:** Experimental data summary of ZnS:Mn-Asp NCs. (ICP-AES: inductively couples plasma atominc emission spectroscopy; PSA: particle size analyzer; DLS: dynamic light scattering.)

Methods	Data
UV-VIS absorption (λ_max_, nm)	320
PL emission (λ_max_, nm)PL efficiency (%)	5909.81
concentration Mn dopant, ICP-AES (%)	1.70
Average Particle Size, HR-TEM (nm)	5.25
Average Particle Size, XRD (nm)	5.41
Zeta Potential, Zeta-PSA (mV)	−4.58
Average size of aggregates in water, DLS (nm)	19.8
